# Innovation in nursing education: point-of-care ultrasound for obtaining difficult venous access

**DOI:** 10.1590/0034-7167-2024-0472

**Published:** 2025-12-08

**Authors:** Fernanda Raphael Escobar Gimenes, Patricia Rezende do Prado, Mayra Gonçalves Menegueti, Mónica Francisca Santana Apablaza

**Affiliations:** I Universidade de São Paulo. Ribeirão Preto, São Paulo, Brazil

**Keywords:** Ultrasonography, Interventional, Catheters, Nursing, Education, Nursing, Teaching, Diploma Programs., Ultrasonografía Intervencional, Catéteres, Enfermería, Enseñanza, Programas de Graduación en Enfermería.

## Abstract

**Objectives:**

to report the experience of introducing ultrasound-guided difficult venous access using point-of-care ultrasound in an undergraduate nursing course.

**Methods:**

a descriptive, experience report type study conducted with fifth-semester nursing students. The course used the Moodle platform, interactive classes with audiovisual resources and quizzes, and hands-on laboratory activities with a virtual simulator, phantom, and physical venous access simulator. Faculty members received specific point-of-care ultrasound training.

**Results:**

organization and integration between theory and practice were observed, with materials available on Moodle, hands-on activities aligned with theoretical content, and individualized feedback. Students reported satisfaction, meaningful learning, and increased confidence in applying the technique.

**Conclusions:**

the inclusion of point-of-care ultrasound in the nursing curriculum proved effective, preparing students for complex clinical scenarios with greater safety. This experience highlights the importance of integrating technologies such as point-of-care ultrasound into nursing education.

## RESUMEN

**Objetivos:**

relatar la experiencia de la introducción de la técnica de acceso venoso difícil guiado por ultrasonido utilizando *point-of-care ultrasound* en una asignatura de pregrado en Enfermería.

**Métodos:**

estudio descriptivo, tipo relato de experiencia, realizado con estudiantes de enfermería del quinto semestre. La asignatura utilizó la plataforma Moodle, clases interactivas con recursos audiovisuales y cuestionarios, y laboratorio *hands-on* con simulador virtual, *phantom* y simulador físico de acceso venoso. Los docentes recibieron capacitación específica en *point-of-care ultrasound*.

**Resultados:**

se observó organización e integración entre teoría y práctica, con materiales disponibles en Moodle, actividades prácticas alineadas con el contenido teórico y retroalimentación individualizada. Los estudiantes relataron satisfacción, aprendizaje significativo y mayor confianza en la aplicación de la técnica.

**Conclusiones:**

la inclusión del *point-of-care ultrasound* en el currículo de enfermería se mostró eficaz, preparando a los estudiantes para escenarios clínicos complejos con mayor seguridad. La experiencia demuestra la importancia de la incorporación de tecnologías como el *point-of-care ultrasound* en la formación de enfermeros.

## INTRODUCTION

The use of point-of-care ultrasound (PoCUS) has become increasingly common in clinical practice, including its use by nurses. The incorporation of advanced techniques, such as PoCUS, into the venous network assessment, the establishment of difficult venous access, and the undergraduate nursing curriculum represents a significant innovation. This approach aims to enhance future professionals’ technical and semiological skills as well as increase confidence and ensure greater patient safety.

The implementation of PoCUS simulation courses for advanced practice nurses in Japan demonstrated significant improvements in image acquisition and interpretation as well as participant confidence. These courses, originally intended for physicians, were adapted for nurses, resulting in notable behavioral changes, such as an increase in the number of ultrasound examinations performed in daily practice^(1)^.

Furthermore, a study highlighted the effectiveness of PoCUS in emergency settings, where generalist and advanced practice nurses were able to perform rapid and accurate assessments of trauma patients. The research demonstrated that the use of PoCUS not only improved clinical assessment accuracy but also reduced critical intervention time, highlighting the importance of this technology in patient care^(2)^.

Another study investigated the impact of PoCUS on central venous catheter insertion in patients with difficult venous access. The results showed that ultrasound guidance significantly increased the first-attempt insertion success rate and reduced complications associated with the procedure. This study reinforced the relevance of PoCUS as an essential tool for invasive nursing procedures^(3)^.

In this regard, the Brazilian National Health Regulatory Agency, an agency created in 1999 and linked to the Ministry of Health, which aims to promote the population’s health, recommended the use of a visualization methodology for intravenous cannulation, in adults and children with difficult intravenous access^(4)^.

The implementation of PoCUS in nursing training programs was also explored in another study, emphasizing the need for a structured curriculum that includes theoretical and practical components. The research indicated that adequate PoCUS training not only improves professionals’ technical skills but also increases confidence and job satisfaction, promoting a safer and more efficient care environment^(5)^.

Experience gained in medical education also offers valuable insights for implementing PoCUS in undergraduate nursing education. Researchers revealed that more than half of medical schools in the United States have already integrated PoCUS into their undergraduate curricula, with many more in the planning stages. The research highlighted the importance of a national consensus on best practices for PoCUS instruction to optimize its effectiveness and promove adoption by institutions that have not yet implemented this technology^(6)^.

Furthermore, research conducted during the COVID-19 pandemic demonstrated the feasibility and effectiveness of teaching PoCUS in both in-person and virtual formats. The results showed that both groups of students-those who attended in-person and virtual sessions-showed significant improvements in their ultrasound skills. This suggests that flexibility in teaching methods can be beneficial, especially during times of physical restrictions^(7)^.

Researchers also emphasized the importance of PoCUS in medical education, noting that including a well-structured PoCUS curriculum can increase fundamental medical knowledge and confidence in ultrasound use. The study suggests that small-group teaching sessions with clear learning objectives and hands-on practice are essential for successful PoCUS teaching^(8)^.

Inspired by these promising results, we decided to introduce the ultrasound-guided technique for difficult intravenous access in the third-year Clinical Nursing course at the *Escola de Enfermagem de Ribeirão Preto, Universidade de São Paulo*. This experience report aims to describe the implementation of this innovative technique in the curriculum and the methods used to teach students.

The introduction of PoCUS into undergraduate nursing education aligns the curriculum with contemporary clinical practices, preparing students to face complex clinical challenges with greater competence and confidence. This report will contribute to existing literature by offering valuable insights into the effectiveness and benefits of incorporating advanced technologies into nursing education.

## OBJECTIVES

To report the experience of introducing the ultrasound-guided difficult intravenous access technique into an undergraduate nursing course.

## METHODS

### Ethical aspects

This study is an experience report focused on describing educational practices without involving the collection of sensitive data or research subjects. Therefore, in accordance with ethical guidelines, approval by the Research Ethics Committee was waived.

### Study design

This is a descriptive, experience report type study carried out at a nursing school linked to a public university in Brazil.

### Study period and place

The experiment took place in the first semester of 2024, in the “Comprehensive Care for Hospitalized Adults and Older Adults with Clinical Conditions” course, taught to fifth-semester bachelor undergraduate students in nursing. The course has a total workload of 150 hours, 30 of which are credited for coursework, and is structured on the university’s Moodle platform, providing an integrated virtual learning environment.

The discipline aims to provide the necessary tools for students to be able to provide nursing care to hospitalized adults and older adults with clinical conditions, based on the development of the Nursing Process stages, such as Assessment, Diagnosis, Planning, Implementation and Evaluation, guided by Horta’s Conceptual Model^(9)^ and Standardized Language Systems (NANDA-I, Inc., Nursing Outcomes Classification and Nursing Interventions Classification)^(10-12)^.

### Participants

The course is taught by a team of eight nursing educators to a group of 80 students, who are divided into two classes of 40 each.

### Procedures

#### Educator training and qualifications

Before introducing the topic into teaching, the course’s faculty members underwent 30 hours of PoCUS training, which included theoretical and practical classes as well as activities on a virtual platform. The lead author is the founder and leader of the Point-of-Care Ultrasound Research and Development Center, the first in Latin America specifically focused on nurses. Our goal is to consolidate our recognition as a reference and center of excellence in research and development in the field of PoCUS. Our mission is to promote continuous learning opportunities and form a community of nurses highly skilled in the use of this technology. It is worth noting that the other faculty members of the course are also members of the center and actively conduct research in this field.

#### Theoretical part in person

The class titled “Nursing Care for Adults and Older Patients with Difficult Intravenous Access and Risk for Vascular Trauma” was taught to two groups of 40 students each. The class structure was divided into two main parts:

The two-hour in-person theoretical portion was conducted through an interactive class, which used real ultrasound images to encourage discussion on key concepts related to the topic. Additionally, illustrative videos were presented, showing ultrasound images obtained with different transducers, providing a more comprehensive understanding of the technique. The content covered during the interactive theoretical class included relevant legislation, purpose of PoCUS for nurses, key concepts (including echogenicity, attenuation, frequency, and image gain), physical principles of ultrasound, anatomical landmarks (with an emphasis on the distinction between veins and arteries), ultrasound-guided access in the short and long axes (patient and transducer preparation, imaging mode selection, vessel assessment, and use of color Doppler), and the scientific evidence supporting this practice. Furthermore, the class was contextualized based on the clinical case below:

*Roberto, a 90-year-old widower, was admitted to the internal medicine unit with a history of chronic kidney disease and was scheduled to begin hemodialysis. Due to this condition, his veins are small and difficult to access. After several failed attempts at venipuncture using the traditional method, the unit nurse decided to proceed with ultrasound-guided venipuncture, based on the identification of the “Risk for vascular trauma” NANDA International, Inc. nursing diagnosis.* (NANDA-I)

Despite the recent exclusion of the “Risk for vascular trauma” diagnosis in the 2024-2026 edition of NANDA-I^(10)^, its application is justified by its clinical relevance and recurrence in healthcare practice, as evidenced in a previous study^(13)^.

During the lecture, quizzes were administered to test students’ knowledge and maintain their engagement in the learning process. After the lecture, flashcards were used as an additional resource to help strengthen the main concepts covered. Post-class quizzes were also administered to assess students’ understanding of the topic.

**Figure 1:**
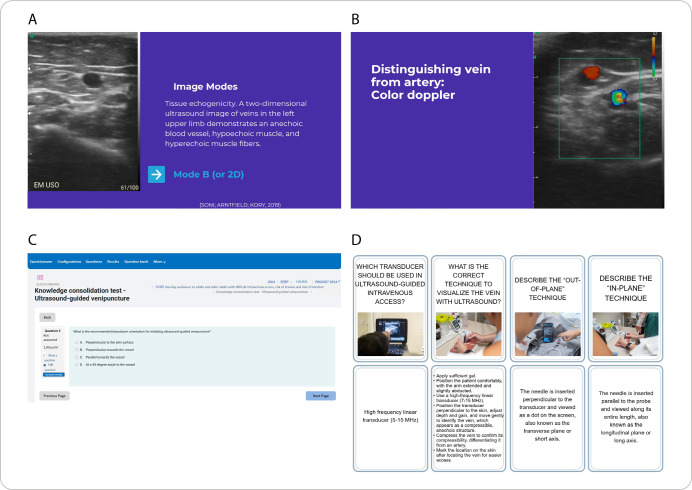
Examples of resources used in the interactive lesson on difficult ultrasound-guided venipuncture, Ribeirão Preto, São Paulo, Brazil, 2024

Figure 1 illustrates the use of the different resources in the interactive lecture.

*A - Use of slides with ultrasound images; B - Use of videos demonstrating real ultrasound images; C - Application of quiz during the interactive class; D - Flashcards to consolidate the main concepts.*

#### Hands-on part

**Figure 2:**
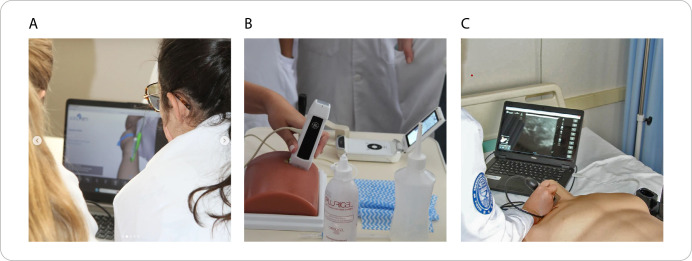
Educational resources used in the hands-on lab, Ribeirão Preto, São Paulo, Brazil, 2024

The class’s hands-on part, with a total workload of four hours per student, was held at the institution’s Nursing Practice Simulation Center. During this phase, students had the opportunity to apply the knowledge acquired in the interactive theoretical class through hands-on laboratory activities. Various resources were used, such as a virtual simulator for ultrasound practice, and low-fidelity simulation using a phantom and a physical simulator for ultrasound-guided venous access (Figure 2).

*A - Virtual simulator for ultrasound-guided puncture training; B - Use of a phantom for ultrasound-guided puncture training; C - Physical simulator for skill training.*

The virtual simulator used in the course is an advanced educational tool designed to facilitate the learning and practice of ultrasound in a safe and controlled environment. Virtual simulators are widely used in medical and, more recently, nursing training programs due to their ability to provide a realistic and interactive learning experience.

The simulator used in our course offers a wide range of training modules covering various areas of ultrasound, including intravenous access. Each module is designed to provide detailed instruction and specific practice for each area of study. The software also includes a vast library of real clinical cases, allowing students to practice interpreting ultrasound images in a variety of clinical scenarios. These cases are designed to simulate situations that healthcare professionals may encounter in daily practice. During training sessions, the simulator provides immediate feedback on user performance, highlighting areas of success and areas for improvement. This feedback is crucial for continuous learning and real-time error correction.

Furthermore, the simulator uses high-quality ultrasound images captured from real patients to ensure students have an accurate and detailed visual experience. This helps develop the ability to recognize and interpret anatomical structures. Additionally, the technological resource allows users to interact with the ultrasound images intuitively, adjusting parameters such as depth, gain, and transducer position. This interactivity simulates the experience of performing a real ultrasound examination.

Finally, the simulator offers remote access, allowing students to practice and review content from anywhere, at any time. This is especially useful for supplementing in-person learning with self-study. For use in the course, students were provided access via login and password.

To organize the hands-on activities, students were divided into groups of up to ten people, and each group rotated between three distinct training stations. The first station was dedicated to the use of the virtual simulator (Figure 2A), allowing students to practice the ultrasound technique in a controlled and safe environment. At the second station, students had the opportunity to practice ultrasound-guided puncture using a five-way phantom (Figure 2B), developing essential practical skills. The third station featured a physical ultrasound-guided intravenous access simulator (Figure 2C), providing a more realistic experience closer to clinical practice.

#### Assessment

The activities were assessed using an electronic form developed in Google Forms. This form contained 27 questions divided into several categories: awareness-raising; lesson development; closure; professor communication; teaching strategy; assessment; and self-assessment. Additionally, a space was provided for suggestions. Each question offered five answer options, ranging from “strongly agree” to “strongly disagree” (supplementary material). The link to access the form was available on the Moodle platform, and completion was not mandatory.

## RESULTS

The assessment of the activity on ultrasound-guided difficult intravenous access was conducted using an electronic form containing 27 questions, categorized as awareness-raising, lesson development, closure, teaching strategies, professor communication, assessment, and self-assessment. Although the response rate was limited (n=2), the students who responded positively assessed all aspects covered. Both students marked “strongly agree” on items such as clarity of objectives, stimulation of interest, connection between theory and practice, and appropriate use of teaching resources such as videos and quizzes. The open-ended comments reinforced this perception, with praise such as “One of the best classes I’ve ever had” and “Very good teaching and explanations”.

In addition, in the final assessment of the course (n=7), students highlighted the integration of theory and practice, the use of simulators, and the focus on clinical reality as the main positive aspects. Qualitative responses revealed feelings of relief, resilience, and gratitude, and highlighted that the course contributed significantly to their professional development, especially in preparing them for challenging clinical settings. Suggestions indicated a desire for more time for extensive content and reinforced the importance of continuous feedback.

These data, although limited in number, indicate a positive perception among students, highlighting the potential of using PoCUS in undergraduate nursing education as an effective and well-accepted pedagogical tool. Data collection in future editions of the course could enhance quantitative analysis robustness.

## DISCUSSION

The experience reported with the “Nursing Care for Adults and Older Patients with Difficult Intravenous Access and Risk for Vascular Trauma” class demonstrated a positive impact on students’ teaching-learning process, especially in the development of clinical reasoning and acquisition of technical skills related to PoCUS. Data collected through student assessments revealed high levels of satisfaction with the pedagogical approach, confirming the potential of the combined use of theoretical, interactive, and simulator resources in teaching advanced nursing techniques. Students particularly highlighted the alignment between theory and practice, the clarity of classes, and the increased confidence in performing the ultrasound-guided venous access procedure.

The prior introduction of the professor training stage was considered essential to ensure the quality of the experience offered. Professor preparation, based on active methodologies and use of simulators, was crucial to the safe and efficient conduct of the activities. This aspect aligns with international recommendations, which highlight professor training as one of the pillars for the successful implementation of PoCUS for training healthcare professionals.

The course’s structure, using virtual simulators, phantoms, and physical models, provided students with hands-on experiences in a safe environment, fostering controlled repetition and trial-and-error learning. Audiovisual resources and quizzes administered during and after the lecture promoted content retention and maintained student engagement, a strategy recognized in the literature as effective in teaching clinical skills.

The spontaneous reports of gratitude, enthusiasm, and a sense of achievement presented by students at the end of the course reinforce the importance of methodologies that prioritize student autonomy and protagonism in the learning process. These perceptions also highlight the transformative role of introducing technologies like PoCUS into nursing undergraduate programs, contributing to the development of more prepared and confident professionals facing complex clinical situations.

Although the “Risk for vascular trauma” nursing diagnosis was recently excluded from the NANDA-I taxonomy (2024-2026)^(10)^, the findings of this experience and specialized literature corroborate its clinical relevance, especially in situations involving multiple venipuncture attempts^(14)^. A recent study demonstrated a significant incidence of peripheral vascular trauma in patients undergoing invasive procedures, which justifies the need for preventive and targeted interventions, such as the use of PoCUS in the Nursing Process^(13)^.

Additionally, the results of this experiment reinforce that, despite being internationally recognized as an effective tool for reducing complications and increasing the safety of vascular procedures, PoCUS still faces barriers to its consolidation in nursing curricula. The lack of specific guidelines, limited technological infrastructure, and the shortage of qualified faculty are obstacles that need to be overcome. The creation of research centers and centers of excellence in PoCUS, such as the one developed by faculty members in this experiment, may represent a promising path to overcoming these gaps and expanding the presence of PoCUS in nursing education and practice.

### Study limitations

Despite its contributions, this experience report has limitations inherent to its descriptive design. The results reflect the experience of a single academic setting, with a limited number of responses to the assessment instruments, which limits the generalizability of the findings. Furthermore, there was no longitudinal follow-up to verify the consolidation of learning or its impact on actual clinical practice. Future studies, with larger samples and analytical designs, could deepen the understanding of the effectiveness of PoCUS in nursing education.

### Contributions to nursing

The use of PoCUS by nurses is feasible, and its incorporation into clinical practice can be encouraged as an important complementary propaedeutic tool, within the Nursing Process, for the development of more targeted and safe nursing diagnoses and nursing interventions.

## CONCLUSIONS

The inclusion of PoCUS teaching for difficult intravenous access in undergraduate nursing courses proved to be an innovative strategy. The experience reported demonstrates that integrating theory and practice, using resources such as virtual simulators, phantoms, and physical simulators, provides students with the development of important skills for safe and efficient clinical practice. Student satisfaction and perception of meaningful learning reinforce the importance of incorporating technologies such as PoCUS into nursing training, preparing them for the challenges of contemporary healthcare and contributing to patient safety.

## Data Availability

The research data are available within the article.
