# Supplementary material for: Innovation in nursing education: point-of-care ultrasound for obtaining difficult venous access
Source: Rev Bras Enferm. 2025 Dec 8;78(Suppl 3):e20240472. doi: 10.1590/0034-7167-2024-0472 (PMC12707843; doi:10.1590/0034-7167-2024-0472)
Supplement: Translation

# Translation: Inovação no ensino de enfermagem: ultrassonografia à beira leito para obtenção de acesso venoso difícil

**DOI:** 10.1590/0034-7167-2024-0472pt

**Authors:** Fernanda Raphael Escobar Gimenes, Patricia Rezende do Prado, Mayra Gonçalves Menegueti, Mónica Francisca Santana Apablaza

**Affiliations:** I Universidade de São Paulo. Ribeirão Preto, São Paulo, Brasil

**Keywords:** Ultrassonografia de Intervenção, Catéter Venoso Periférico, Enfermagem, Ensino, Programas de Graduação em Enfermagem.

## Abstract

**Objetivos:**

relatar a experiência da introdução da técnica de acesso venoso difícil guiado por ultrassom usando *point-of-care ultrasound* em disciplina de graduação em enfermagem.

**Métodos:**

estudo descritivo, do tipo relato de experiência, realizado com estudantes de enfermagem do 5º período. A disciplina utilizou plataforma *Moodle*, aula interativa com recursos audiovisuais e *quizzes*, e laboratório *hands-on* com simulador virtual, fantoma e simulador físico de acesso venoso. Docentes receberam treinamento específico em *point-of-care ultrasound*.

**Resultados:**

observaram-se organização e integração entre teoria e prática, com materiais disponíveis no *Moodle*, atividades práticas alinhadas ao conteúdo teórico e *feedback* individualizado. Alunos relataram satisfação, aprendizado significativo e maior confiança na aplicação da técnica.

**Conclusões:**

a inclusão do *point-of-care ultrasound* no currículo de enfermagem se mostrou eficaz, preparando os estudantes para cenários clínicos complexos com maior segurança. A experiência demonstra a importância da inserção de tecnologias como a *point-of-care ultrasound* na formação de enfermeiros.

## INTRODUÇÃO

A utilização da ultrassonografia à beira do leito, conhecida como *point-of-care ultrasound* (PoCUS), tem se tornado cada vez mais comum na prática clínica, abrangendo também seu uso por enfermeiros. A incorporação de técnicas avançadas, como a PoCUS, na avaliação da rede venosa, na obtenção de acesso venoso difícil e no currículo de graduação em enfermagem representa uma inovação relevante. Essa abordagem visa aprimorar as habilidades técnicas e semiológicas dos futuros profissionais, assim como aumentar a confiança e garantir maior segurança para os pacientes.

A implementação de cursos de simulação para o uso de PoCUS, no Japão, para enfermeiros de prática avançada demonstrou melhorias significativas na aquisição e interpretação de imagens, bem como na confiança dos participantes. Esses cursos, originalmente destinados a médicos, foram adaptados para os enfermeiros, resultando em mudanças comportamentais notáveis, como o aumento do número de exames ultrassonográficos realizados na prática diária^(1)^.

Além disso, estudo destacou a eficácia da PoCUS em ambientes de emergência, onde enfermeiros generalistas e de prática avançada foram capazes de realizar avaliações rápidas e precisas em pacientes traumatizados. A pesquisa demonstrou que a utilização da PoCUS não só melhorou a precisão na avaliação clínica, mas também reduziu o tempo para intervenções críticas, evidenciando a importância dessa tecnologia no cuidado ao paciente^(2)^.

Outro estudo investigou o impacto da PoCUS na inserção de cateteres venosos centrais em pacientes com acesso venoso difícil. Os resultados mostraram que a orientação por ultrassom aumentou significativamente a taxa de sucesso na primeira tentativa de inserção, além de reduzir complicações associadas ao procedimento. Este estudo reforçou a relevância da PoCUS como ferramenta essencial para procedimentos invasivos em enfermagem^(3)^.

Neste sentido, a Agência Nacional de Vigilância Sanitária, autarquia criada em 1999 e vinculada ao Ministério da Saúde, que tem a finalidade de promover a saúde da população, recomendou a utilização de metodologia de visualização para instalação de cateteres venosos, em adultos e crianças, com rede venosa difícil^(4)^.

A implementação da PoCUS em programas de treinamento para enfermeiros também foi explorada em outra investigação, enfatizando a necessidade de um currículo estruturado que inclua componentes teóricos e práticos. A pesquisa indicou que a formação adequada em PoCUS não só melhora as habilidades técnicas dos profissionais, mas também aumenta a confiança e satisfação no trabalho, promovendo um ambiente de cuidado mais seguro e eficiente^(5)^.

A experiência adquirida no ensino médico também oferece *insights* valiosos para a implementação da PoCUS no ensino de graduação em enfermagem. Pesquisadores revelaram que mais da metade das escolas de medicina nos Estados Unidos já integrou a PoCUS em seus currículos de graduação, com muitas outras em fase de planejamento. A pesquisa destacou a importância de um consenso nacional sobre as melhores práticas para instrução da PoCUS, a fim de otimizar sua eficácia e facilitar a adoção por parte das instituições que ainda não implementaram essa tecnologia^(6)^.

Além disso, pesquisa conduzida durante a pandemia de COVID-19 demonstrou a viabilidade e eficácia do ensino de PoCUS tanto em formatos presenciais quanto virtuais. Os resultados mostraram que ambos os grupos de estudantes, aqueles que participaram de sessões presenciais e aqueles que participaram de sessões virtuais, apresentaram melhorias significativas em suas habilidades de ultrassonografia. Isso sugere que a flexibilidade no método de ensino pode ser benéfica, especialmente em tempos de restrições físicas^(7)^.

Pesquisadores também enfatizaram a importância da PoCUS na educação médica, destacando que a inclusão de um currículo bem estruturado de PoCUS pode aumentar o conhecimento médico fundamental e a confiança no uso do ultrassom. O estudo sugere que sessões de ensino em pequenos grupos, com objetivos de aprendizagem claros e prática *hands-on*, são características essenciais para o sucesso do ensino de PoCUS^(8)^.

Inspiradas por esses resultados promissores, decidimos introduzir a técnica de acesso venoso difícil guiado por ultrassom na disciplina “Enfermagem Clínica” do terceiro ano dos cursos de graduação da Escola de Enfermagem de Ribeirão Preto da Universidade de São Paulo. Este relato de experiência tem como objetivo descrever a implementação dessa técnica inovadora no currículo e os métodos utilizados para ensinar os alunos.

A introdução da PoCUS no ensino de graduação em enfermagem alinha o currículo com as práticas clínicas contemporâneas, preparando os alunos para enfrentar desafios clínicos complexos com maior competência e segurança. Este relato contribuirá para a literatura existente, oferecendo *insights* valiosos sobre a eficácia e os benefícios da incorporação de tecnologias avançadas no ensino de enfermagem.

## OBJETIVOS

Relatar a experiência da introdução da técnica de acesso venoso difícil guiado por ultrassom em disciplina de graduação em enfermagem.

## MÉTODOS

### Aspectos éticos

Este estudo é um relato de experiência focado na descrição de práticas educacionais sem envolver coleta de dados sensíveis ou sujeitos de pesquisa. Portanto, conforme diretrizes éticas, a aprovação pelo Comitê de Ética em Pesquisa foi dispensada.

### Desenho do estudo

Estudo descritivo, do tipo relato de experiência, realizado em escola de enfermagem vinculada à universidade pública no Brasil.

### Período e local de estudo

A experiência ocorreu no primeiro semestre de 2024, na disciplina “Cuidado Integral ao Adulto e Idoso Hospitalizados em Situação Clínica”, ministrada para os estudantes do quinto período do curso de graduação bacharelado em enfermagem. A disciplina contém carga horária total de 150 horas, sendo 30 destinadas à crédito trabalho, e é estruturada na plataforma *Moodle* da universidade, proporcionando um ambiente virtual de aprendizagem integrado.

A disciplina tem como objetivo proporcionar as ferramentas necessárias para que o estudante seja capaz de prestar assistência de enfermagem ao adulto e ao idoso hospitalizados em situação clínica, a partir do desenvolvimento das etapas do Processo de Enfermagem, como Avaliação de Enfermagem, Diagnóstico de Enfermagem, Planejamento de Enfermagem, Implementação de Enfermagem e Evolução de Enfermagem, à luz do Modelo Conceitual de Horta^(9)^ e dos Sistemas de Linguagens Padronizadas (NANDA-I, Inc., Classificação de Resultados de Enfermagem e Classificação de Intervenções de Enfermagem)^(10-12)^.

### Participantes

A disciplina é ministrada por uma equipe de oito docentes para um grupo composto por 80 estudantes, os quais são divididos em duas turmas de 40 cada.

### Procedimentos

#### Formação e qualificação docente

Antes de introduzir o tema no ensino, as docentes da disciplina foram submetidas a treinamento em PoCUS com duração de 30 horas, que incluiu aulas teóricas e práticas, além de atividades em uma plataforma virtual. A autora principal é a fundadora e líder do Centro de Pesquisa e Desenvolvimento em Ultrassonografia *Point-of-Care*, o primeiro da América Latina voltado especificamente para enfermeiros. Nosso objetivo é consolidar o reconhecimento como referência e centro de excelência em pesquisa e desenvolvimento na área da PoCUS. Nossa missão é promover oportunidades de aprendizado contínuo e formar uma comunidade de enfermeiros altamente capacitados no uso dessa tecnologia. Ressalta-se que as demais docentes da disciplina também integram o centro e desenvolvem pesquisas ativas nesse campo de investigação.

#### Parte teórica presencial

A aula intitulada “Assistência de Enfermagem ao Adulto e Idoso com Acesso Venoso Difícil e Risco de Trauma Vascular” foi ministrada para duas turmas, cada uma composta por 40 estudantes. A estrutura da aula foi dividida em duas partes principais:

A parte teórica presencial, com duração de duas horas, foi conduzida por meio de uma aula interativa, na qual foram utilizadas imagens ultrassonográficas reais para estimular a discussão sobre os principais conceitos relacionados ao tema. Além disso, vídeos ilustrativos foram apresentados, mostrando imagens ultrassonográficas obtidas com diferentes transdutores, proporcionando uma compreensão mais abrangente da técnica. Os conteúdos abordados durante a aula teórica interativa incluíram legislações pertinentes, finalidade da PoCUS para os enfermeiros, principais conceitos (incluindo ecogenicidade, atenuação, frequência e ganho de imagem), princípios físicos do ultrassom, marcos anatômicos (com ênfase na distinção entre veias e artérias), acesso guiado por ultrassom nos eixos curto e longo (preparo do paciente e do transdutor, seleção do modo de imagem, avaliação do vaso, e uso do doppler colorido), e as evidências científicas que sustentam tal prática. Outrossim, a aula foi contextualizada a partir do caso clínico abaixo:

*Roberto, 90 anos, viúvo, foi admitido na unidade de clínica médica com histórico de doença renal crônica; está programado o início de hemodiálise. Devido a esta condição, suas veias apresentam calibre reduzido e dificuldade de acesso. Após várias tentativas falhas de punção venosa utilizando o método tradicional, o enfermeiro da unidade decide proceder com a punção venosa guiada por ultrassom, a partir da identificação do diagnóstico de enfermagem “Risco de trauma vascular” da NANDA International, Inc.* (NANDA-I)

Apesar da exclusão recente do diagnóstico “Risco de trauma vascular” na edição 2024-2026 da NANDA-I^(10)^, sua aplicação se justifica pela relevância clínica e recorrência na prática assistencial, conforme evidenciado em estudo pregresso^(13)^.

Durante a aula teórica, foram aplicados *quizzes* para testar o conhecimento dos estudantes e manter seu engajamento no processo de aprendizagem. Após a aula, *flashcards* foram utilizados como recurso adicional para auxiliar na sedimentação dos principais conceitos abordados. *Quizzes* pós-aula também foram aplicados para avaliar a compreensão dos estudantes sobre o tema.

A Figura 1 ilustra o uso dos diferentes recursos na aula interativa.

**Figura 1:** Exemplos de recursos empregados na aula interativa sobre punção venosa difícil guiada por ultrassom, Ribeirão Preto, São Paulo, Brasil, 2024

*A - Uso de slides com imagens ultrassonográficas; B - Uso de vídeos que demonstram imagens ultrassonográficas reais; C - Aplicação de quiz durante a aula interativa; D - Flashcards para sedimentação dos principais conceitos.*

#### Parte prática

A parte prática da aula, com carga horária total de quatro horas por aluno, foi realizada no Centro de Simulação de Práticas de Enfermagem da instituição. Nessa etapa, os estudantes tiveram a oportunidade de aplicar os conhecimentos adquiridos na aula teórica interativa por meio de atividades *hands-on* em laboratório. Foram utilizados diferentes recursos, como o simulador virtual, para a prática de ultrassonografia, e simulação de baixa fidelidade, com o uso de fantoma e simulador físico de acesso venoso guiado por ultrassom (Figura 2).

**Figura 2:** Recursos educacionais utilizados no laboratório *hands-on*, Ribeirão Preto, São Paulo, Brasil, 2024

*A - Simulador virtual para o treino da punção guiada por ultrassom; B - Uso de fantoma para o treino da punção guiada por ultrassom; C - Simulador físico para o treino da habilidade.*

O simulador virtual utilizado na disciplina é uma ferramenta educacional avançada projetada para facilitar o aprendizado e a prática de ultrassonografia em um ambiente seguro e controlado. Simuladores virtuais são amplamente utilizados em programas de treinamento médico e, mais recentemente, de enfermagem, devido à sua capacidade de oferecer uma experiência de aprendizado realista e interativa.

O simulador utilizado em nossa disciplina oferece uma ampla gama de módulos de treinamento que cobrem diversas áreas da ultrassonografia, incluindo acesso venoso. Cada módulo é desenvolvido para fornecer instruções detalhadas e práticas específicas para cada área de estudo. O *software* também inclui uma vasta biblioteca de casos clínicos reais, permitindo que os estudantes pratiquem a interpretação de imagens ultrassonográficas em cenários clínicos variados. Esses casos são projetados para simular situações que os profissionais de saúde podem encontrar na prática diária. Durante as sessões de treinamento, o simulador fornece *feedback* imediato sobre o desempenho do usuário, destacando áreas de acerto e pontos que necessitam de melhoria. Esse *feedback* é crucial para o aprendizado contínuo e correção de erros em tempo real.

Ademais, o simulador utiliza imagens ultrassonográficas de alta qualidade, capturadas de pacientes reais, para garantir que os estudantes tenham uma experiência visual precisa e detalhada. Isso ajuda a desenvolver a habilidade de reconhecer e interpretar estruturas anatômicas. Outrossim, o recurso tecnológico permite que os usuários interajam com as imagens ultrassonográficas de maneira intuitiva, ajustando parâmetros como profundidade, ganho e posição do transdutor. Essa interatividade simula a experiência de realizar um exame ultrassonográfico real.

Por fim, o simulador oferece a possibilidade de acesso remoto, permitindo que os estudantes pratiquem e revisem os conteúdos de qualquer lugar, a qualquer momento. Isso é especialmente útil para complementar o aprendizado presencial com estudo autônomo. Para seu uso na disciplina, foi disponibilizado o acesso aos estudantes por meio de *login* e senha.

Para a organização das atividades práticas, os estudantes foram divididos em grupos de até dez pessoas, e cada grupo rotacionou entre três estações de treinamento distintas. A primeira estação foi dedicada ao uso do simulador virtual (Figura 2A), permitindo que os estudantes praticassem a técnica de ultrassonografia em um ambiente controlado e seguro. Na segunda estação, os estudantes tiveram a oportunidade de treinar a punção guiada por ultrassom utilizando um fantoma de cinco vias (Figura 2B), desenvolvendo habilidades práticas essenciais. A terceira estação contou com um simulador físico de acesso venoso guiado por ultrassom (Figura 2C), proporcionando uma experiência mais realista e próxima da prática clínica.

#### Avaliação

A avaliação das atividades foi realizada por meio de um formulário eletrônico desenvolvido no *Google Forms*. Este formulário continha 27 questões distribuídas em várias categorias: sensibilização; desenvolvimento da aula; encerramento; comunicação docente; estratégia de ensino; avaliação; e autoavaliação. Além disso, foi disponibilizado um espaço para inclusão de sugestões. Cada pergunta oferecia cinco alternativas de resposta, variando de “concordo totalmente” a “discordo totalmente” (material suplementar). O *link* de acesso ao formulário foi disponibilizado na plataforma *Moodle*, e seu preenchimento não era obrigatório.

## RESULTADOS

A avaliação da atividade sobre acesso venoso difícil guiado por ultrassom foi realizada por meio de formulário eletrônico contendo 27 questões, categorizadas em sensibilização, desenvolvimento da aula, encerramento, estratégias de ensino, comunicação docente, avaliação e autoavaliação. Embora a taxa de resposta tenha sido limitada (n=2), os alunos que responderam avaliaram positivamente todos os aspectos abordados. Ambos marcaram “concordo totalmente” em itens como clareza dos objetivos, estímulo ao interesse, conexão entre teoria e prática, e uso adequado dos recursos didáticos, como vídeos e *quizzes*. Os comentários livres reforçaram essa percepção, com destaque para elogios como “Uma das melhores aulas que tive” e “Didática e explicação muito boa”.

Além disso, na avaliação final da disciplina (n=7), os alunos apontaram como principais pontos positivos a integração entre teoria e prática, o uso de simuladores e o foco na realidade clínica. As respostas qualitativas revelaram sentimentos de alívio, superação e gratidão, e destacaram que a disciplina contribuiu significativamente para a formação profissional, especialmente no preparo para contextos clínicos desafiadores. As sugestões indicaram o desejo de mais tempo para conteúdos extensos e reforçaram a importância do *feedback* contínuo.

Esses dados, embora limitados em número, indicam uma percepção positiva por parte dos estudantes, evidenciando o potencial do uso da PoCUS no ensino de graduação em enfermagem como uma ferramenta pedagógica eficaz e bem aceita. A coleta de dados em futuras edições da disciplina poderá ampliar a robustez da análise quantitativa.

## DISCUSSÃO

A experiência relatada com a aula “Assistência de Enfermagem ao Adulto e Idoso com Acesso Venoso Difícil e Risco de Trauma Vascular” demonstrou impacto positivo no processo de ensino-aprendizagem dos estudantes, especialmente no desenvolvimento do raciocínio clínico e na aquisição de habilidades técnicas relacionadas à PoCUS. Os dados coletados por meio da avaliação discente evidenciaram altos níveis de satisfação com a proposta pedagógica, confirmando potencial do uso combinado de recursos teóricos, interativos e simuladores no ensino de técnicas avançadas em enfermagem. Os alunos destacaram, em especial, o alinhamento entre teoria e prática, a clareza na condução das aulas e o aumento da confiança ao realizarem o procedimento de acesso venoso guiado por ultrassom.

A introdução prévia da etapa de formação docente foi considerada essencial para garantir a qualidade da experiência oferecida. O preparo das professoras, realizado com base em metodologias ativas e uso de simuladores, foi determinante para a condução segura e eficiente das atividades. Esse aspecto se alinha às recomendações internacionais, que apontam a capacitação docente como um dos pilares para a implementação bem-sucedida da PoCUS na formação de profissionais de saúde.

A estrutura da disciplina, com uso de simuladores virtuais, fantomas e modelos físicos, proporcionou aos alunos vivências práticas em um ambiente seguro, favorecendo a repetição controlada e aprendizado por tentativa e erro. Os recursos audiovisuais e *quizzes* aplicados durante e após a aula teórica favoreceram a retenção do conteúdo e mantiveram o engajamento dos estudantes, estratégia reconhecida na literatura como eficaz no ensino de competências clínicas.

Os relatos espontâneos de gratidão, entusiasmo e sentimento de superação apresentados pelos alunos ao final da disciplina reforçam a importância de metodologias que priorizam a autonomia e o protagonismo do estudante no processo de aprendizagem. Tais percepções também evidenciam o papel transformador da introdução de tecnologias como a PoCUS na graduação em enfermagem, contribuindo para a formação de profissionais mais preparados e confiantes diante de situações clínicas complexas.

Embora o diagnóstico de enfermagem “Risco de trauma vascular” tenha sido recentemente excluído da taxonomia da NANDA-I (2024-2026)^(10)^, os achados desta experiência e a literatura especializada corroboram sua relevância clínica, especialmente em situações envolvendo múltiplas tentativas de punção venosa^(14)^. Estudo recente demonstrou incidência significativa de traumatismos vasculares periféricos em pacientes submetidos a procedimentos invasivos, o que justifica a necessidade de intervenções preventivas e direcionadas, como o uso da PoCUS no Processo de Enfermagem^(13)^.

Além disso, os resultados desta experiência reforçam que, apesar de reconhecida internacionalmente como uma ferramenta eficaz para reduzir complicações e aumentar a segurança dos procedimentos vasculares, a PoCUS ainda encontra barreiras para sua consolidação nos currículos de enfermagem. A ausência de diretrizes específicas, a limitação de infraestrutura tecnológica e a escassez de docentes habilitados são obstáculos que precisam ser superados. A formação de núcleos de pesquisa e centros de excelência em PoCUS, como o desenvolvido pelas docentes desta experiência, pode representar um caminho promissor para superar essas lacunas e ampliar a presença da PoCUS no ensino e prática de enfermagem.

### Limitações do estudo

Apesar das contribuições, este relato de experiência apresenta limitações inerentes ao seu desenho descritivo. Os resultados refletem a vivência de um único cenário acadêmico, com número reduzido de respostas aos instrumentos de avaliação, o que restringe a generalização dos achados. Ademais, não houve seguimento longitudinal para verificar a consolidação do aprendizado ou seu impacto na prática clínica real. Estudos futuros, com amostras maiores e delineamentos analíticos, poderão aprofundar a compreensão sobre a efetividade da PoCUS na formação em enfermagem.

### Contribuições para a área da enfermagem

O uso da PoCUS por enfermeiros é factível, e a sua incorporação na prática clínica pode ser estimulada como importante ferramenta propedêutica complementar, dentro do Processo de Enfermagem, para elaboração de diagnósticos de enfermagem e intervenções de enfermagem mais direcionadas e seguras.

## CONCLUSÕES

A inclusão do ensino da PoCUS para obtenção de acesso venoso difícil em disciplinas de graduação em enfermagem se mostrou uma estratégia inovadora. A experiência relatada demonstra que a integração entre teoria e prática, utilizando recursos como simuladores virtuais, fantomas e simuladores físicos, proporciona aos estudantes o desenvolvimento de habilidades importantes para a prática clínica segura e eficiente. A satisfação dos alunos e a percepção de um aprendizado significativo reforçam a importância da inserção de tecnologias como a PoCUS na formação de enfermeiros, preparando-os para os desafios da assistência à saúde contemporânea e contribuindo para a segurança do paciente.

## Data Availability

Os dados da pesquisa estão disponíveis no corpo do artigo.
